# Association between a vascular endothelial growth factor gene polymorphism (rs2146323) and diabetic retinopathy: a meta-analysis

**DOI:** 10.1186/s12886-015-0155-3

**Published:** 2015-11-09

**Authors:** Ying Zeng, Fangjie Dai, Ke Yang, Yongqing Tang, Meng Xu, Yiwu Zhou

**Affiliations:** Department of Ophthalmology of the Shanghai Tenth People’s Hospital, and Laboratory of Clinical Visual Sciences of Tongji Eye Institute, and Department of Regenerative Medicine, Tongji University School of Medicine, Shanghai, China; Institute of Cardiovascular Disease, Ruijin Hospital, Shanghai Jiaotong University School of Medicine, Shanghai, China; Department of Development, Great China Region of Novartis, Shanghai, China

**Keywords:** Vascular endothelial growth factor, Polymorphism, Meta-analysis, Diabetic retinopathy

## Abstract

**Background:**

Vascular endothelial growth factor (VEGF) is thought to play an important role in the pathogenesis of diabetic retinopathy (DR). Previous studies have associated the *VEGF* rs2146323 polymorphism with the risk of DR. However, the results of these studies are inconsistent. A meta-analysis was performed to evaluate the association between the *VEGF* rs2146323 polymorphism and the risk of DR.

**Methods:**

The PubMed, EMBASE, Web of Science and Google Scholar literature databases until March 2015 were searched. The differences in the studies were expressed in the form of an odds ratio (OR) and the corresponding 95 % confidence interval (CI). Heterogeneity among the studies was tested using the I^2^ statistic based on the Q test.

**Results:**

A total of four studies (598 cases and 709 controls) were included in the meta-analysis. A significant association was found involving the rs2146323 polymorphism in the dominant model (CA + AA VS. CC) (OR = 1.38, CI = 1.10–1.72, *P* = 0.005) and the co-dominant model (CA VS. CC) (OR = 1.37, CI = 1.08–1.74, *P* = 0.008).

**Conclusions:**

Our meta-analysis confirmed the association between the *VEGF* rs2146323 polymorphism and the risk of DR.

## Background

Diabetic retinopathy (DR) is considered one of the most common microvascular complications in diabetes. It leads to blindness among adults aged 20–74 years and is regarded as the main cause of blindness in people with diabetes [1]. Although the mechanisms of DR remain largely unknown, increasing evidence has implicated both genetics and environmental factors in the pathogenesis of DR [2, 3].

VEGF is expressed and secreted from vascular endothelial cells [4], Müller cells [5], astrocytes [6], retinal pigment epithelial cells (RPE) [7], and ganglion cells [8]; it exerts its physiological effects by mediating vascular permeability, angiogenesis, endothelial cell growth, cell migration, and apoptosis [9]. Increased levels of VEGF in the blood and the retina are linked to the pathogenesis of DR [10]. Moreover, Anti-VEGF therapy is currently recommended for patients with diabetic macular edema, a very specific subtype of DR [11].

The human *VEGF* gene is located on chromosome 6p21.3 with 7 introns and 8 exons and shows polymorphism [12]. Many *VEGF* SNPs have been reported showing association with DR, such as rs699947 [13], rs833061 [14], rs3025039 [15], which have been assessed quantitatively by meta-analyses [16–18]. But we noted that rs2146323 have been reported showing inconsistent association with DR [19–24]. Therefore, we conducted this meta-analysis.

A meta-analysis is a statistical procedure of pooling the data from individual studies, increasing the effective sample size, and producing a single estimate of an effect [25]. The aim of our study was to evaluate the association between the *VEGF* rs2146323 polymorphism and DR by performing a meta-analysis.

## Methods

### Literature search and inclusion criteria

All the literature in PubMed, EMBASE, Web of Science, Google Scholar literature databases was searched. The search included only English language publications before the deadline of the March 1st, 2015. To search all of the literature related to the rs2146323 polymorphism, we used the following search strategies: (vascular endothelial growth factor or VEGF or VEGFA or “vascular endothelial growth factor A/genetics”[mesh]) and (diabetic retinopathy or diabetes retinopathy or DR or “diabetic retinopathy/genetics’[mesh] or “macular edema/genetics”[mesh]). We chose the literature that involved the polymorphism based on the title and abstract of the articles and even the full text when necessary. The meta-analysis was performed for cases with any form of DR compared with diabetic without retinopathy (DWR). After removal of the duplicates, we used the following inclusion criteria to filter the articles: (1) the study was a case–control study; (2) definitive methods were applied to the diagnosis of diabetic retinopathy, including the Early Treatment of Diabetic Retinopathy Study (ETDRS) and the guidelines of the Expert Committee Report of the American Diabetes Association (DR includes proliferative diabetic retinopathy (PDR) and non-proliferative diabetic retinopathy (NPDR)); (3) the VEGF rs2146323 polymorphism was evaluated; (4) the sample size was sufficient for the calculation of the odds ratio (OR) with 95 % confidence interval (CI); and (5) the control group followed Hardy-Weinberg equilibrium (HWE; a *χ*^2^ value of Pearson’s chi-square test > 3.84 (df = 1) indicates deviation from HWE). The most recent article will be used to extract data if the authors published more than one article with the same study data.

### Data extraction and quality assessment

The data were independently extracted by two reviewers and both of the reviewers’ results were submitted to a third reviewer who verified the results. If there were inconsistencies, the three reviewers discussed the disagreements to resolve the differences. The following items were extracted: (1) the name of the first author; (2) the year of publication; (3) the origin country; (4) the racial descent of the study population; (5) the sample size of the cases and controls; (6) duration of diabetes; (7) gender ration (male, %); (8) HbA1c level; (9) the genotype frequency; (10) the genotyping methods; and (11) the HWE (for the studies in which there was no HWE assessment, we calculated this parameter) [26]. The Newcastle–Ottawa Scale (NOS) tools, which were recommended by Cochrane, were used to evaluate the quality of the eligible studies, like a previous study [27].

### Statistical analysis

The six genetics models were used for analyses: dominant model: CA + AA vs. CC; allele model: A vs. C; recessive model: AA vs. CA + CC; co-dominant model: CA vs. CC; co-dominant model means AA vs. CC; over-dominant model means CA vs. CC + AA; additive model: CC vs. AA [25]. The differences in the studies were expressed in the form of the odds ratio (OR) and the corresponding 95 % confidence interval (CI). The pooled OR was evaluated in 6 genetic models. The pooled OR was determined using a Z test (PZ < 0.05 was considered statistically significant). Heterogeneity among studies was tested by the I^2^ statistic based on Q test. Studies with I^2^ < 50 % or *P* > 0.10 were considered to be of low heterogeneity, and the fixed effects model was used to calculated the pooled OR, otherwise, random effects model was used to pool OR. STATA software (version 12.0; STATA Corporation, College Station, TX, USA) was used for the analyses. Two-sided *P* < 0.05 was considered statistically significant.

## Results

### Characteristics of the studies

**Fig. 1:**
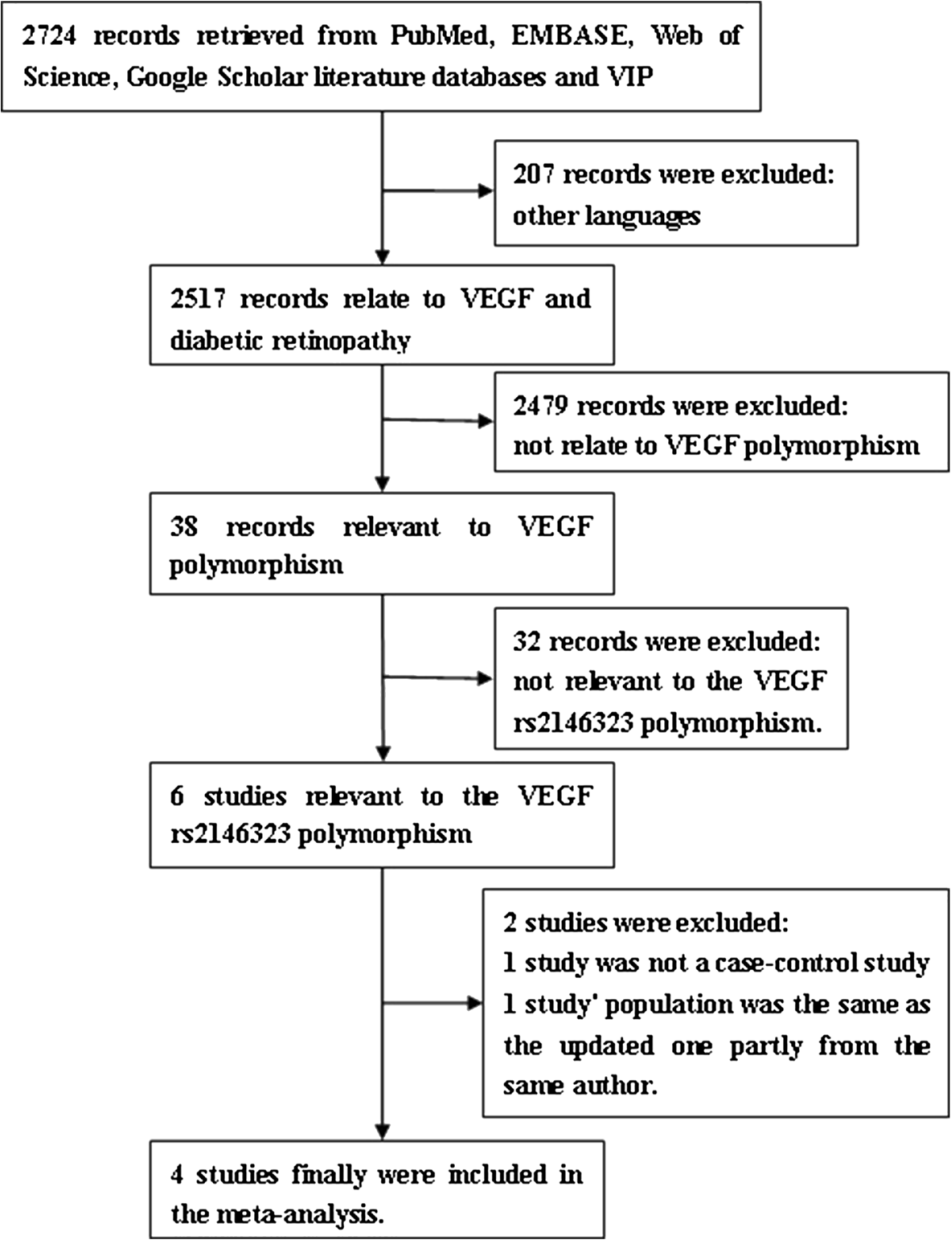
Flow chart of the inclusion and exclusion of the studies

A total of 2724 articles were retrieved in the initial search. Of these, 207 articles were written in a non-English language, 2479 articles were irrelevant regarding the *VEGF* polymorphism in DR, and 32 articles were irrelevant regarding the *VEGF* rs2146323 polymorphism were excluded based on reading the titles or abstracts. In addition, 2 articles were excluded because one was not a case–control study [24] and one was a study previously reported by same author concerning the same population [23]. The flow chart describing the inclusion and exclusion of the studies is shown in Fig. 1, and the characteristics of the included studies are listed in Table 1. The characteristics and the quality assessment of eligible studies are listed in Table 1. Generally, the quality of included studies was moderate. The major design deficiencies of eligible studies were as follows: 1) the authors did not report whether the subjects were consecutively enrolled; 2) confounding factors were not well controlled; and 3) non-response rates were not reported.

**Table 1:** Characteristics and quality assessment of the included studies

Author	Year	Ethnicity	Country	N	Diabetes	Duration of diabetes (DR/DWR), years	Male (DR/DWR), %	HbA1c (DR/DWR), %	Genotype typing	NOS
Yang XF [22]	2014	Asian	China	490	Type 2	15 ± 5 / 13 ± 7	0.45 / 0.39	7.8 ± 1.7 / 7.0 ± 1.4	PCR-RFLP	5
Kangas-Kontio T [21]	2009	Caucasian	Finland	224	Type 1/2	24 ± 10 / 25 ± 7	0.60 / 0.51	Not reported	RT-PCR/Taqman	6
Abhary S [19]	2009	Caucasian	Australia	487	Type 1	31 ± 13 / 15 ± 9	0.39 / 0.48	8.8 ± 2.3 / 7.6 ± 2.5	Mass spectrometer	5
					Type 2	18 ± 9 / 13 ± 9	0.56 / 0.65	7.5 ± 3.4 / 6.6 ± 2.9		
Churchil AJ [20]	2008	Caucasian	UK	106	Type 1/2	23 / 25	0.60 / 0.52	Not reported	PCR-RFLP	5

*DR* diabetes with retinopathy, *DWR* diabetes without retinopathy, *PCR RFLP* PCR with restriction fragment length polymorphism, *HWE* Hardy-Weinberg equilibrium, *NOS* The Newcastle–Ottawa Scale [23]. Values are the mean ± standard deviation, where appropriate

### Pooled effects analyses

**Fig. 2:**
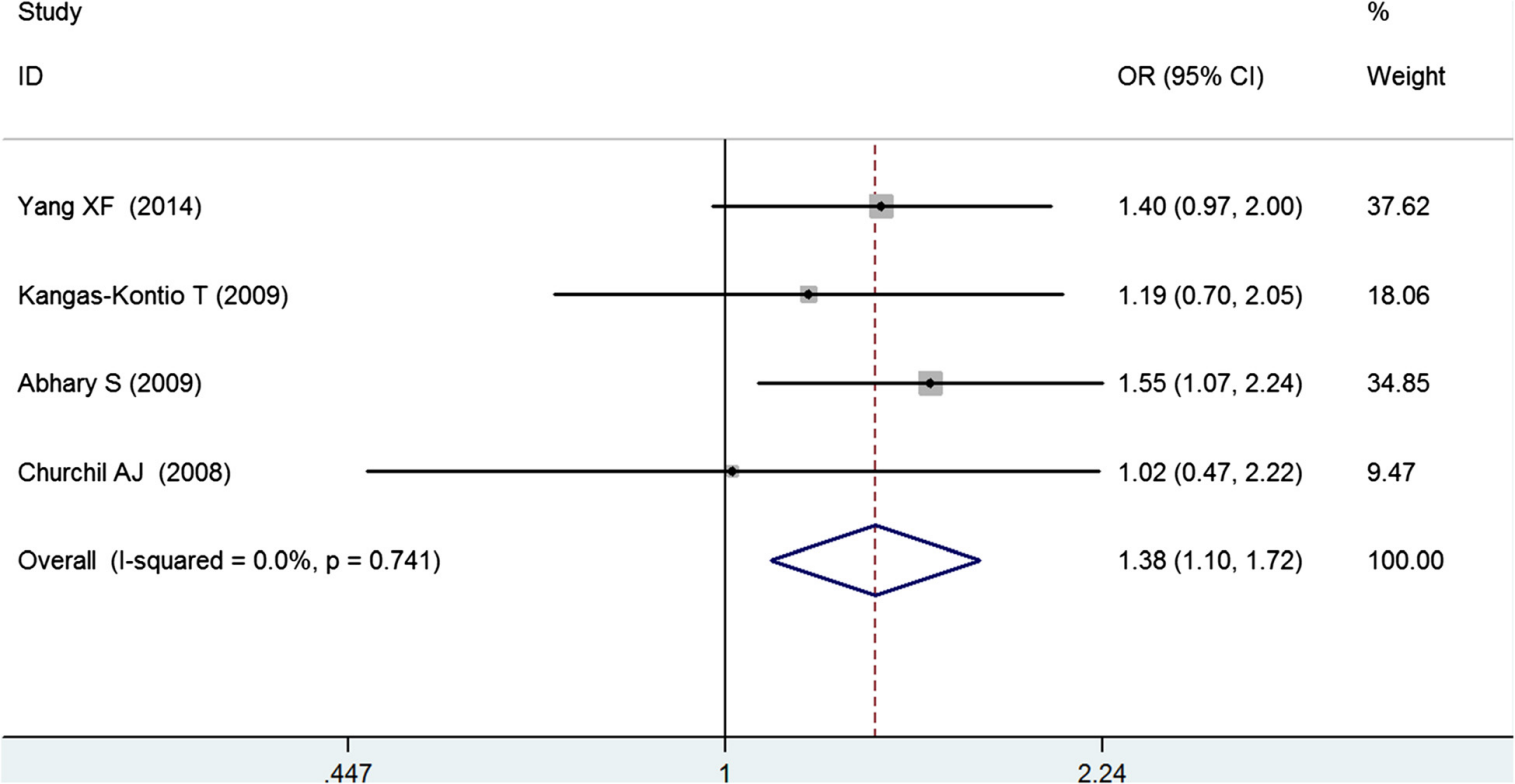
Meta-analysis of the association between the *VEGF* rs2146323 polymorphism and diabetic retinopathy in the dominant model (CA + AA vs. CC)

**Fig. 3:**
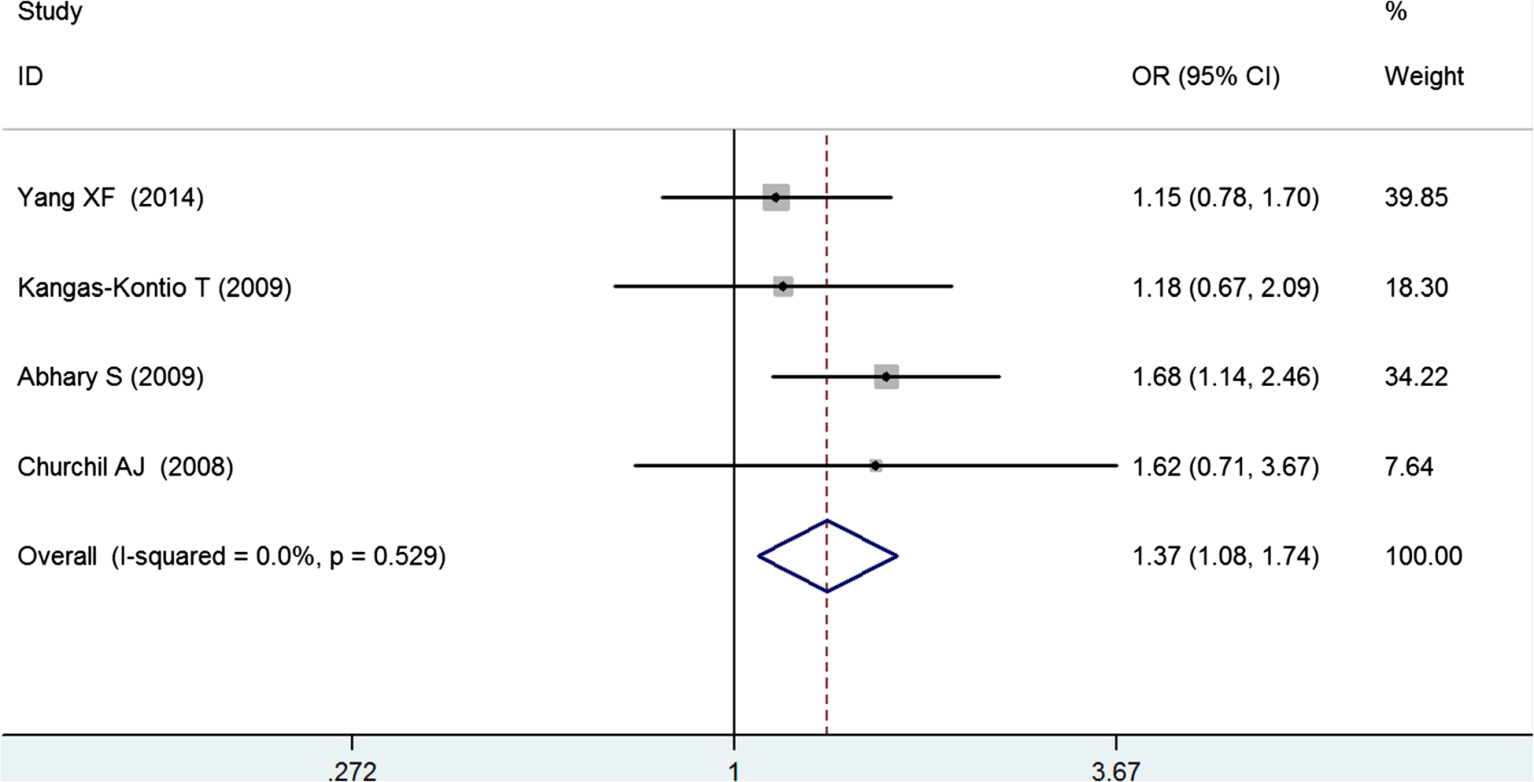
Meta-analysis of the association between the *VEGF* rs2146323 polymorphism and diabetic retinopathy in the co-dominant model (CA vs. CC)

A total of 598 diabetes with retinopathy (DR) cases and 709 diabetes without retinopathy (DWR) controls were identified (Table 2), and 6 genetic models were performed for analyzing the association between the rs2146323 polymorphism and DR. Our results showed a significant association between the rs2146323 polymorphism and DR in the dominant model (CA + AA VS CC) (OR = 1.38, CI = 1.10–1.72, PZ = 0.005, PH = 0.741) (Fig. 2) and in the co-dominant model (CA VS CC) (OR = 1.37, CI = 1.08–1.74, PZ = 0.008, PH = 0.529) (Fig. 3). No significant heterogeneity was found between the two models. In the genetic models with significant association, the Caucasian subgroups were consistent with the overall patient population (dominant model, OR = 1.37, CI = 1.03–1.81, PZ = 0.031, PH = 0.538; co-dominant model, OR = 1.52, CI = 1.13–2.04, PZ = 0.006, PH = 0.601). However, no significant association was found in the Chinese subgroup (dominant model, OR = 1.40, PZ = 0.070; co-dominant model CA VS CC, OR = 1.15, PZ = 0.470) (Table 2).

**Table 2:** Genotype frequency of VEGF rs2146323 polymorphism

Author	DR/DWR	DWR			DR			HWE (*χ*2)
		CC	CA	AA	CC	CA	AA	
Yang XF [22]	214/276	112	72	30	167	93	16	0.4
Kangas-Kontio T [21]	127/97	47	61	19	40	44	13	0.03
Abhary S [19]	212/275	77	113	22	129	113	33	1.13
Churchil AJ [20]	45/61	19	26	0	26	22	13	3.64

**Table 3:** Pooled ORs and 95 % CIs of the association between the *VEGF* rs2146323 polymorphism and DR in six models

Model	Contrasts	Number of study	Pooled method	OR (95 % CI)	Z	Pz	Heterogeneity	
							I^2^	P
Dominant CA + AA vs. CC	Chinese	1	Fixed-effect	1.40 (0.97–2.00)	1.81	0.070		
	Caucasian	3		1.37 (1.03–1.81)	2.16	0.031	0.0 %	0.538
	Overall	4		1.38 (1.10–1.72)	2.82	0.005	0.0 %	0.741
Allele A vs. C	Chinese	1	Random-effect	1.52 (1.14–2.03)	2.88	0.004		
	Caucasian	3		1.03 (0.75–1.43)	0.19	0.847	52.1 %	0.124
	Overall	4		1.16 (0.88–1.53)	1.02	0.306	59.9 %	0.058
Recessive AA vs. CA + CC	Chinese	1	Random-effect	2.65 (1.40–5.00)	3.01	0.003		
	Caucasian	3		0.73 (0.29–1.85)	0.67	0.504	63.9 %	0.063
	Overall	4		1.04 (0.43–2.53)	0.09	0.928	77.5 %	0.004
Co-dominant CA vs. CC	Chinese	1	Fixed-effect	1.15 (0.78–1.71)	0.72	0.470		
	Caucasian	3		1.52 (1.13–2.04)	2.75	0.006	0.0 %	0.601
	Overall	4		1.37 (1.08–1.74)	2.63	0.008	0.0 %	0.529
Co-dominant AA vs. CC	Chinese	1	Random-effect	2.80 (1.46–5.37)	3.09	0.002		
	Caucasian	3		0.89 (0.35–2.24)	0.25	0.801	59.0 %	0.087
	Overall	4		1.26 (0.56–2.84)	0.55	0.585	70.8 %	0.016
Over-dominant CA vs. CC + AA	Chinese	1	Random-effect	1.00 (0.68–1.46)	0.01	0.991		
	Caucasian	3		1.55 (1.09–2.21)	2.41	0.016	29.1 %	0.244
	Overall	4		1.36 (0.96–1.91)	1.74	0.081	51.2 %	0.105
Additive CC vs. AA	Chinese	1	Random-effect	0.36 (0.19–0.69)	3.09	0.002		
	Caucasian	3		1.10 (0.46–2.66)	0.22	0.828	55.2 %	0.107
	Overall	4		0.79 (0.36–1.76)	0.57	0.567	69.8 %	0.019

### Risk evaluation of publication bias and sensitive analyses

**Fig. 4:**
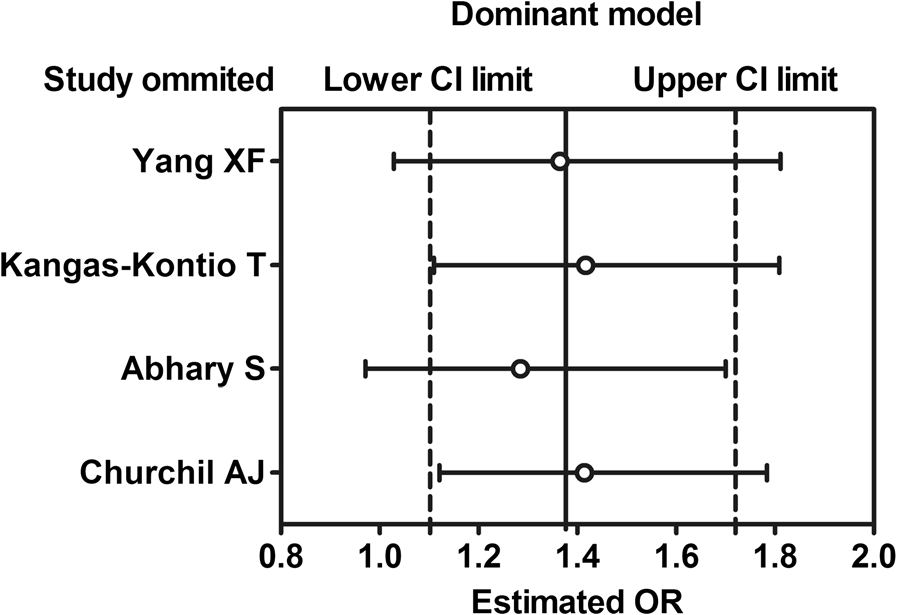
Sensitivity analysis in the dominant model (CA + AA vs. CC)

**Fig. 5:**
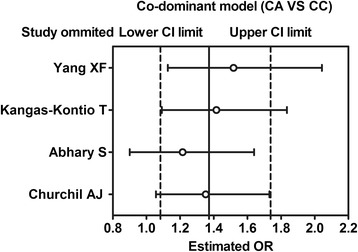
Sensitivity analysis in the dominant model (CA vs. CC)

We presented a basic symmetry with disperse and uniform points in Begg’ funnel plot. Further statistical analysis show that there were no publication bias in the inclusive studies (dominant model: Begg’ test *P* = 0.308, Egger’s test *P* = 0.083; allele model: Begg’ test *P* = 0.308, Egger’s test *P* = 0.145; recessive model: Begg’ test *P* = 0.734, Egger’s test *P* = 0.387; co-dominant model CA VS CC: Begg’ test *P* = 1.000, Egger’s test *P* = 0.968; co-dominant model AA VS CC: Begg’ test *P* = 0.734, Egger’s test *P* = 0.272; over-dominant model: Begg’ test *P* = 0.734, Egger’s test *P* = 0.595; additive model: Begg’ test *P* = 0.734, Egger’s test *P* = 0.272). We conducted the sensitivity analysis, and results show that the pooled OR was close to the total pooled OR and was within the total 95 % CI, which indicated that the results of sensitive analyses for these studies were reliable (Figs. 4 and 5).

## Discussion

The present meta-analysis confirmed the association between the *VEGF* rs2146323 polymorphism and DR. Churchil et al. [20] first reported that the +5092 C/A (rs2146323) polymorphism was significantly associated with proliferative diabetic retinopathy (PDR) in a Caucasian population with type 1 or 2 diabetes, although the sample size for analyzing this polymorphism was relatively small. However, subsequent studies showed inconsistent results. Abhary et al. [19] investigated whether the rs2146323 polymorphism was significantly associated with blinding DR in type 1 but not in type 2 diabetic patients. Kangas-kontio et al. [21] reported no association between rs2146323 and DR. Yang XF et al. [22] first reported a signification association in Chinese type 2 diabetic patients. Considering that the data are not sufficient, we could not exclude the correlation between the type of diabetes and the results. The results from Caucasian populations were consistent with the overall results. However, because only one study in the Chinese population and three studies in the Caucasian population were investigated, further studies on this polymorphism might involve additional data that includes the population, type of DR, and more complicated genetic or environmental background.

To date, the mechanism of the involvement of VEGF in DR development remains unclear. VEGF plays a crucial role in DR by increasing the vascular permeability and neovascularization [4]. Beyond the present study, *VEGF* polymorphisms are significantly associated with the risk of DR [28–30]. The significant *VEGF* gene polymorphism promotes *VEGF* gene expression and increases the levels of VEGF in the serum [31] and the vitreous of the eyes of diabetic patients [32]. However, we could not analyze the rs2146323 and VEGF levels in both blood and the vitreous of diabetic eyes because of insufficient direct data on VEGF expression levels. VEGF +5092 C/A is located in 111-bp 5′ of exon 3, where it is distant from the predicted transcription factor binding sites and is not predicted to be involved in exon splicing alterations [20]. Thus, this makes it more effective for unveiling the mechanism of this polymorphism on VEGF expression and function.

There are several limitations of this meta-analysis. First, the sample size is relatively small because only four studies were included in the meta-analysis. Second, the effects of the type of diabetes on the association between the polymorphism and DR were not addressed because the primary studies did not provide the relevant data. Third, in an attempt to overcome the confounding results of undetectable population stratification, we limited the case and control groups to include only Caucasian ethnicity. We found that the Caucasian subgroups were consistent with the overall patient population. However, there are several uncontrolled confounding factors (age, sex, and duration of diabetes, blood glucose level, medication use, and other complications). Fourth, the present studies did not provide the physical and biochemical examination results, which are useful for interpreting the mechanism of the polymorphism in DR. Finally, although DR cases were classified by fundus photographs or OCT, level of DR has been not presented, which may be the source of heterogeneity.

## Conclusions

In conclusion, our meta-analysis results indicated that there was a significant association between the VEGF rs2146323 polymorphism and the risk of DR. Since most of these polymorphisms are in strong linkage disequilibrium, genome-wide association study (GWAS) can provide more genome-wide genetic association with DR.
